# Hexyl-Aminolevulinate Ethosomes: a Novel Antibiofilm Agent Targeting Zinc Homeostasis in Candida albicans

**DOI:** 10.1128/spectrum.02438-22

**Published:** 2022-10-27

**Authors:** Yingzhe Wang, Wei Long, Feiyin Zhang, Meimei Zhang, Kang Zeng, Xiaoliang Zhu

**Affiliations:** a Department of Dermatology, Nanfang Hospital, Southern Medical University, Guangzhou, China; Universidade de Sao Paulo

**Keywords:** *Candida albicans*, biofilms, hexyl-aminolevulinate, ethosomes, zinc

## Abstract

Substantial drug resistance afforded by Candida albicans biofilms results in ineffective treatment with conventional drugs and persistent infection. Our previous study showed that hexyl-aminolevulinate ethosomes (HAL-ES) act against C. albicans biofilms and weaken their drug resistance and pathogenicity; however, the mechanism involved remains unclear. Here, we systematically evaluated the effects and mechanisms of HAL-ES on biofilm formation and drug resistance. We found that, in addition to mediating antifungal photodynamic therapy, HAL-ES inhibited the early, developmental, and mature stages of biofilm formation compared with fluconazole, HAL, or ES. Notably, adhesion and hyphal formation were significantly inhibited by postdrug effects even after brief exposure (2 h) to HAL-ES. Its therapeutic effect in vivo also has been demonstrated in cutaneous candidiasis. RNA sequencing and quantitative PCR showed that HAL-ES inhibited ribosome biogenesis by disrupting zinc homeostasis in C. albicans, thereby reducing the translation process during protein synthesis. Furthermore, HAL-ES downregulated the expression of multidrug resistance genes and increased fluconazole susceptibility in C. albicans. Our findings provide a novel and efficient method for the treatment of biofilm resistance in C. albicans infection as well as a basis for the application of HAL-ES. We also describe a new strategy for the treatment of biofilm-related infections via zinc restriction.

**IMPORTANCE** Candida albicans is the most prevalent fungal species of the human microbiota. The medical impact of C. albicans on its human host depends on its ability to form biofilms. The intrinsic resistance conferred by biofilms to conventional antifungal drugs makes biofilm-based infections a significant clinical challenge. In this study, we demonstrate the attenuating effect of HAL-ES on C. albicans biofilm formation and drug resistance. Furthermore, we propose that HAL-ES inhibits protein translation by disrupting zinc homeostasis in C. albicans. This study not only provides a novel and effective therapeutic strategy against C. albicans biofilm but also proposes a new strategy to resolve C. albicans biofilm infection by disrupting zinc homeostasis.

## INTRODUCTION

Candida albicans causes candidiasis—a fungal infection of the skin or mucosal membranes—and occurs in symbiotic association with the host’s usual microflora (1). When the body’s microbiota is out of balance and the immune system is compromised, localized and even invasive candidiasis occurs commonly, with mortality rates of up to 50% in immunocompromised patients (2). C. albicans is the most common and difficult-to-treat pathogenic agent of candidiasis, not only because of its heightened drug resistance but also due to biofilm formation, which is an enhanced mode of drug resistance. C. albicans is more prone to biofilm formation than other *Candida* species; indeed, more than 80% of infections are associated with biofilms (3). C. albicans biofilm is a three-dimensional network structure composed of yeast, hyphae, and an extracellular matrix. This structure is highly resistant to antifungal drugs, even at dosages 1,000-fold higher than recommended, hindering the therapeutic effect of antibiotics (4). Consequently, there is an urgent need to develop novel therapeutic approaches against C. albicans biofilms.

In previous research (5), we prepared a highly effective nanodrug, hexyl-aminolevulinate ethosome (HAL-ES), to combat C. albicans biofilms. It not only conferred the pharmaceutical advantages of small particle size, strong permeability, and high stability, but also effectively inhibited the growth of biofilms and reduced their drug resistance and pathogenicity after a single treatment. We hypothesized that the effectiveness of HAL-ES stems from the multiple antibiofilm targets of its two components (6).

HAL is a lipophilic photosensitizer with antibacterial properties generally used in bladder cancer diagnostics (7–9). HAL-mediated antifungal photodynamic therapy (aPDT) leads to reactive oxygen species production and antimicrobial activity without inducing drug resistance (10). ES constitutes a novel nanovesicle delivery system composed of relatively high concentrations of ethanol (20 to 45%), phospholipid bilayers, and water (11). This system has the advantages of highly efficient encapsulation, small particle size, strong permeability, high stability, and low irritation (12, 13). The ethanol content in ES contributes to its antimicrobial activity (14). Therefore, the multifaceted antifungal potential of HAL-ES renders it active in multiple stages of biofilm formation, including yeast adhesion in early dispersal stages, hyphal transition and invasion in mid-developmental stages, and three-dimensional structure formation in late mature stages (4).

In this study, we aimed to systematically explore the effects and mechanism of action of HAL-ES against C. albicans biofilm formation and drug resistance, as well as verify its effectiveness *in vivo*. Our findings provide a novel and efficient strategy for the treatment of fungal infections exhibiting biofilm-related drug resistance and recurrence.

## RESULTS

### HAL-ES promoted the antifungal activity of ES and increased the solubility of HAL.

**FIG 1:**
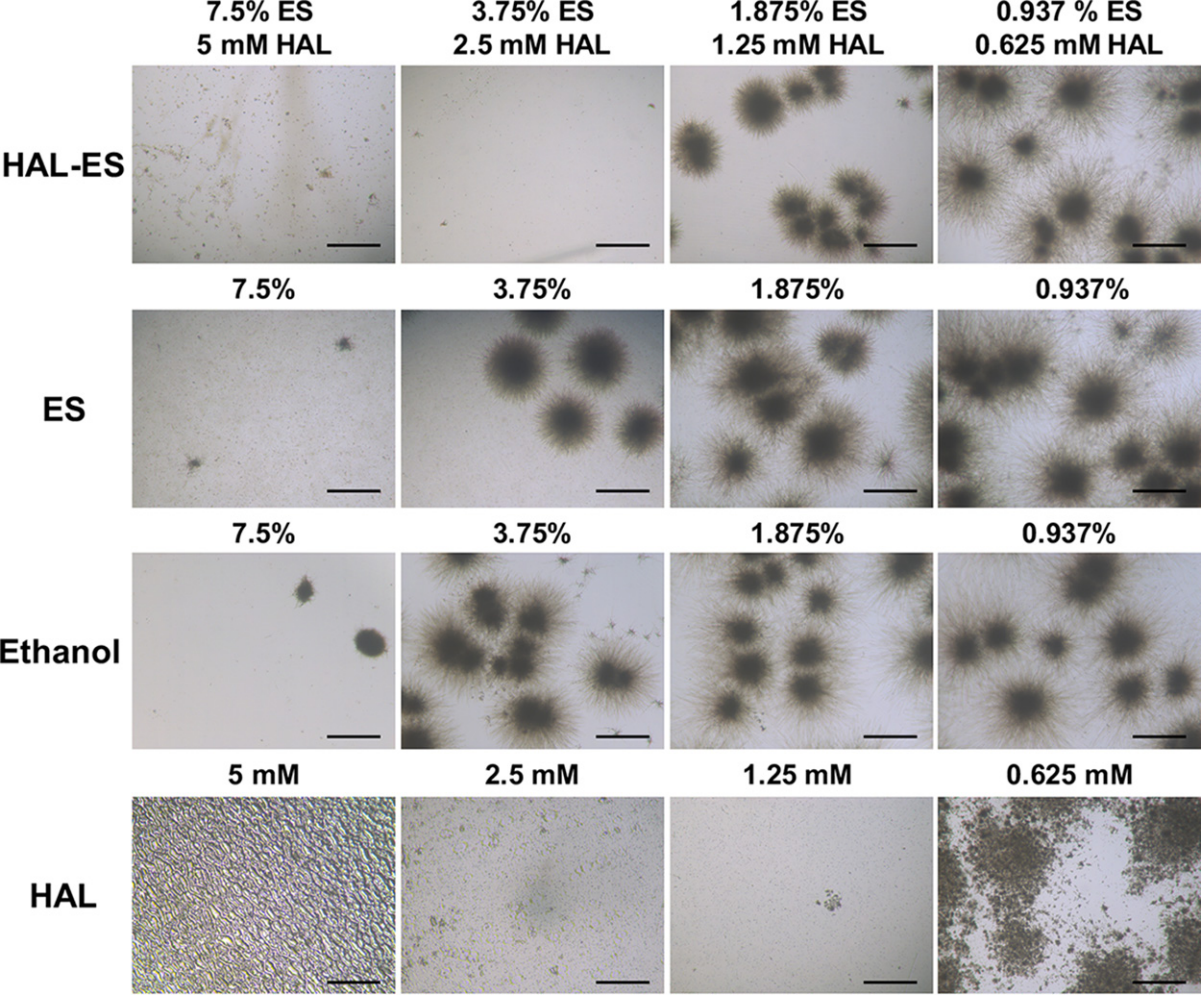
Observations of Candida albicans after 36 h of drug coincubation. Scale bar, 500 μm.

The MIC and minimum fungicidal concentration (MFC) were 7.5% and 15%, respectively, for both ethanol and ES, whereas the MIC and MFC of HAL-ES were both 3.75%, indicating that the addition of HAL increased the antifungal effect of ES (Table 1). ES and ethanol had the same MIC and MFC, but at 7.5% and 3.75%, ES had a slightly more pronounced inhibitory effect on hyphal colony formation (Fig. 1). At 5 mM, HAL reacts with certain components of Roswell Park Memorial Institute (RPMI) 1640 medium, resulting in an insoluble precipitate, possibly due to its instability at a high pH (15). Nonetheless, the addition of 7.5% ES increased the solubility of 5 mM HAL in RPMI 1640 medium, and the MIC of HAL-ES was higher than that of HAL.

**TABLE 1:** MICs and MFCs of different drugs against Candida albicans

Drug	MIC	MFC
HAL-ES	3.75% ES and 2.5 mM (625 μg/mL) HAL	3.75% ES and 2.5 mM (625 μg/mL) HAL
ES	7.5%	15%
Ethanol	7.5%	15%
HAL	1.25 mM (312.5 μg/mL)	2.5 mM (625 μg/mL)
Fluconazole	0.5 μg/mL	>2,048 μg/mL

**FIG 2:**
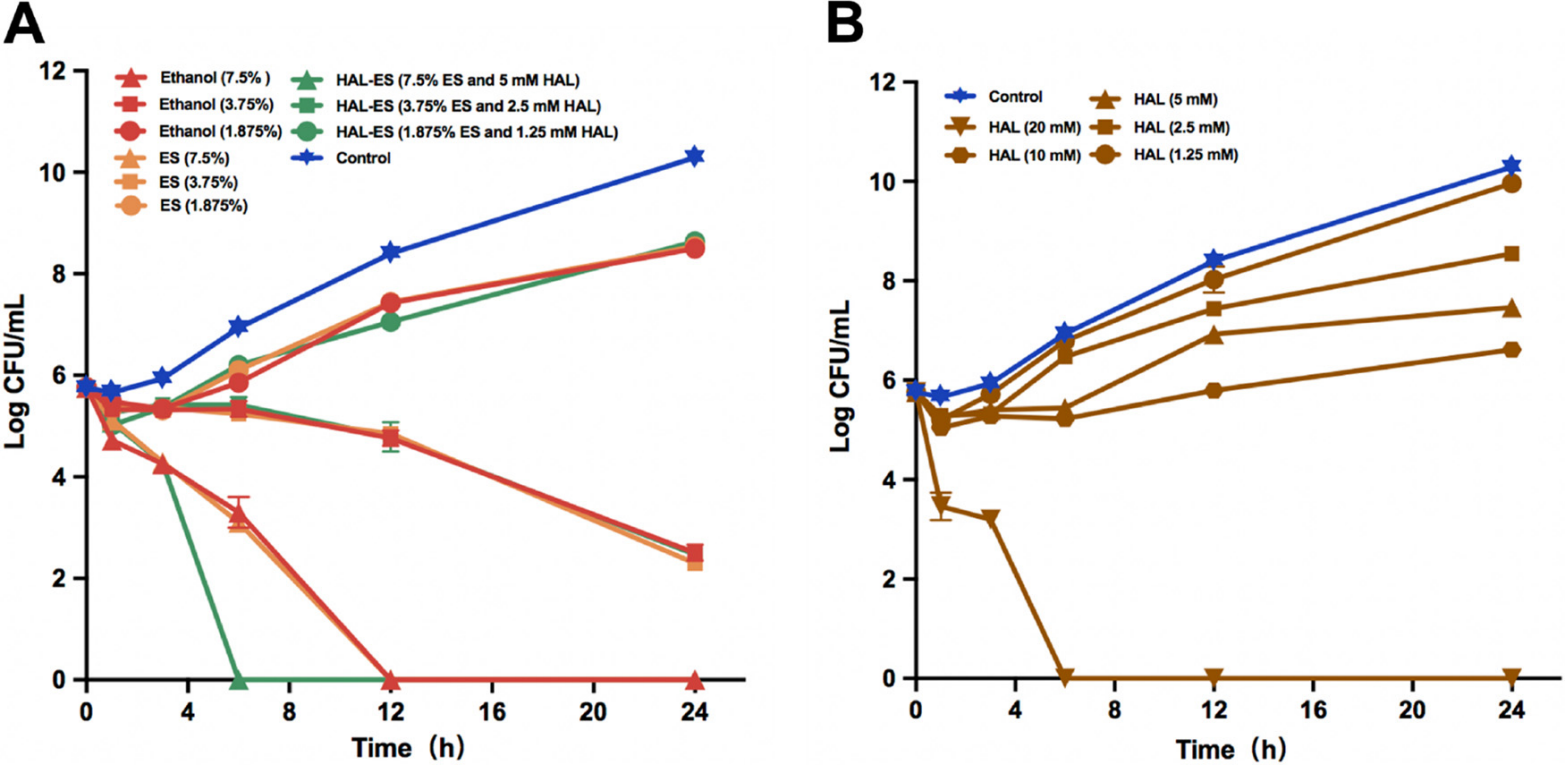
Effects of different drugs on the time-kill curve of Candida albicans. C. albicans cells were incubated in YPD medium containing HAL-ES, ES, or ethanol (A) or HAL (B). Experiments were conducted at 37°C with constant shaking (200 rpm).

We assessed the dynamic antifungal effects of the drugs by using time-kill curves (Fig. 2). For yeast in yeast extract-peptone-dextrose (YPD) medium, the time-kill curves of ethanol, ES, and HAL-ES treatments were similar at concentrations of 3.75% and 1.875%. However, at a concentration of 7.5%, HAL-ES (7.5% ES and 5 mM HAL) exhibited a fungicidal effect within 6 h of incubation, twice as fast as 7.5% ethanol or 7.5% ES alone. An ethanol concentration of >3.75% had a killing effect, while a concentration of 1.875% only inhibited growth. No survival was observed after 6 h of incubation with 20 mM HAL, and growth inhibition was noted at 2.5 to 10 mM HAL.

### HAL-ES impaired adhesion of C. albicans (early dispersal stage of biofilm formation).

**FIG 3:**
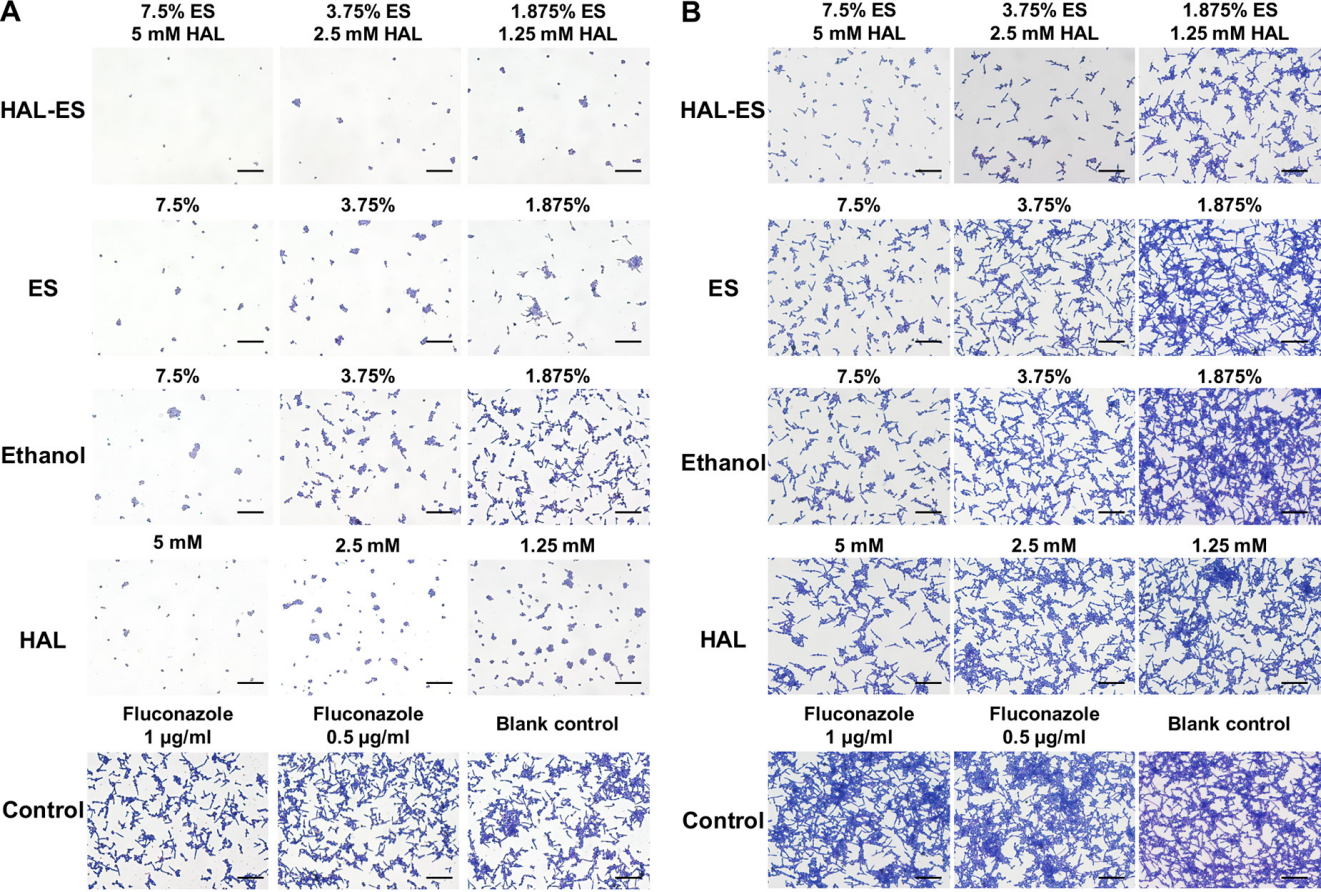
Impairment of early adhesion during biofilm formation. (A) Adhesion capacity of Candida albicans cells in drug-containing medium. (B) Adhesion capacity of drug-exposed C. albicans cells. Scale bar, 50 μm.

Adhesion is the first process in biofilm formation, and it influences the distribution of C. albicans in the biofilm (4). Compared with that of fluconazole at the MIC, the adhesion of C. albicans was inhibited by coincubation with HAL-ES, HAL, ES, or ethanol in a concentration-dependent manner. Additionally, HAL-ES showed the greatest inhibitory effect (Fig. 3A). Adhesion was inhibited even after brief exposure to HAL-ES for 2 h, indicating an antiadherence postexposure effect (Fig. 3B).

### HAL-ES inhibited the yeast-to-hyphal phenotypic transition (mid-developmental stage of biofilm formation).

**FIG 4:**
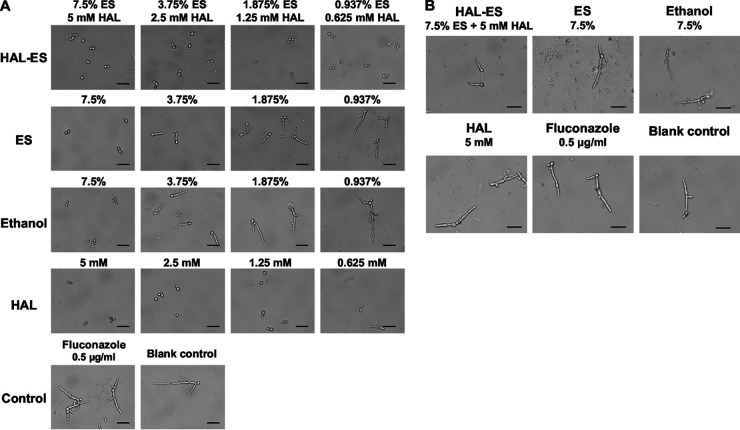
Inhibition of the phenotypic switch from yeast to hyphal form during biofilm development. (A) Level of yeast-to-hyphae transition in drug-containing medium. (B) Level of yeast-to-hyphae transition in drug-exposed Candida albicans cells. Scale bar, 25 μm.

The phenotypic transition of C. albicans is necessary for the development of biofilms. Hyphal formation is another determinant of virulence in invasive tissues (4). Under hypha-inducing conditions, HAL-ES, HAL, ES, or ethanol inhibited hyphal formation and elongation in a concentration-dependent manner compared with fluconazole. Moreover, HAL-ES showed the greatest inhibitory effect (Fig. 4A). A brief incubation of C. albicans with HAL-ES for 2 h inhibited hypha formation, indicating an antihyphal postexposure effect (Fig. 4B).

### HAL-ES inhibited the three-dimensional structure formation of biofilms (late mature stage of biofilm formation).

**FIG 5:**
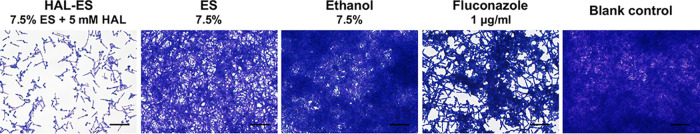
Impairment of biofilm maturation after 24 h of drug incubation. Scale bar, 50 μm.

After coincubation with C. albicans for 24 h, HAL-ES inhibited the formation of a mature three-dimensional biofilm structure by inhibiting elongation of the hyphae compared with fluconazole, ES, and ethanol treatments. The biofilm formed in the ES group lacked the density observed in the ethanol and control groups (Fig. 5).

### HAL-ES promoted the sensitivity of C. albicans to fluconazole.

**FIG 6:**
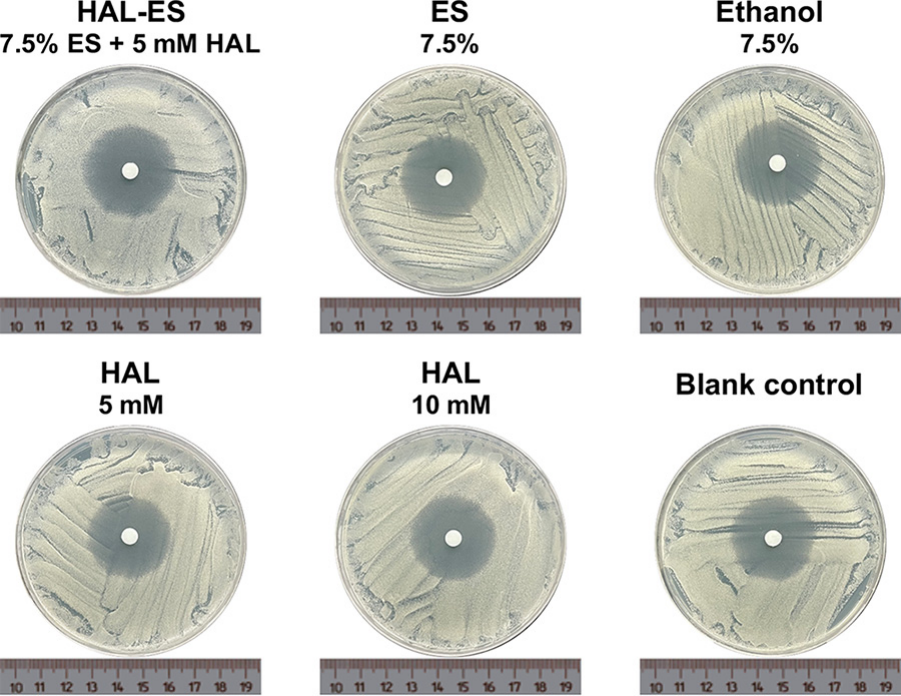
Susceptibility of drug-exposed Candida albicans to fluconazole.

After exposure to HAL-ES for 2 h, the susceptibility of C. albicans to fluconazole increased significantly, while ethanol, ES, or HAL alone had no effect (Table 2 and Fig. 6).

**TABLE 2:** Susceptibility of drug-exposed Candida albicans to fluconazole

Drug	Zone of inhibition (mm)^*a*^
HAL-ES (7.5% ES, 5 mM HAL)	36.33 ± 0.76***
ES (7.5%)	32.1 ± 0.36
Ethanol (7.5%)	31.67 ± 0.57
HAL (5 mM)	31.7 ± 0.72
HAL (10 mM)	32.23 ± 0.31
Blank control	31.6 ± 0.36

a Data are means ± SD (*n* = 3). ***, *P *< 0.001 compared with the blank control group.

### Antifungal effects of aPDT on C. albicans.

**FIG 7:**
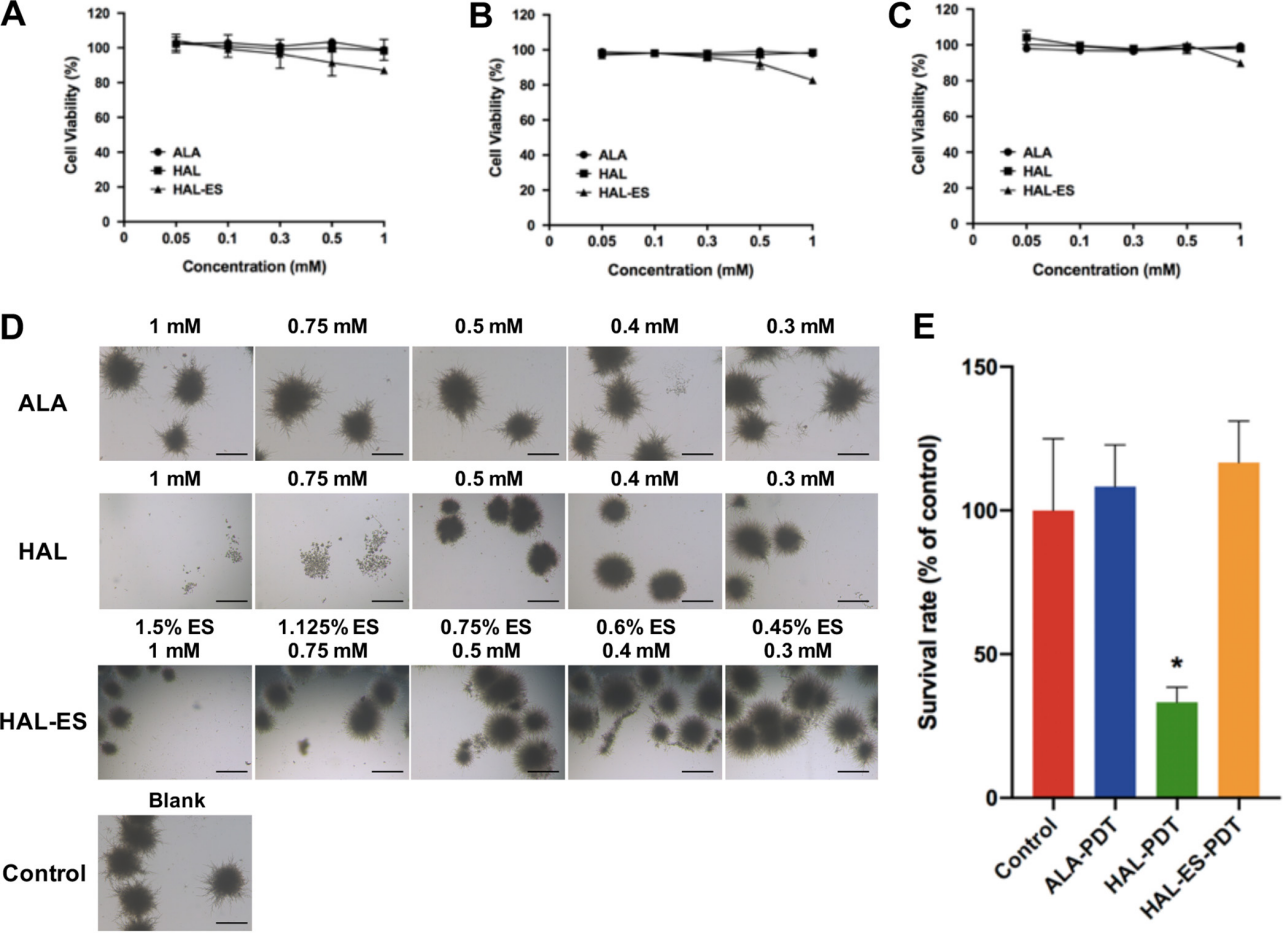
aPDT effect of photosensitizers on Candida albicans. (A to C) Log-transformed cell viability of C. albicans incubated with ALA, HAL, or HAL-ES in YPD medium for 4 h (A), 12 h (B), and 24 h (C). (D) C. albicans cells were incubated with ALA, HAL, or HAL-ES in RPMI 1640 medium for 24 h. Scale bar, 500 μm. (E) aPDT effect mediated by ALA, HAL, or HAL-ES. Data are means ± SD (*n* = 3). *, *P < *0.05 compared with the control group.

We assessed the antifungal effects of aminolevulinate (ALA), HAL, and HAL-ES incubated with C. albicans in YPD and RPMI 1640 medium in the dark for 24 h and found that they had no antifungal effects at concentrations below 300 μM (Fig. 7). We next assessed the effects of aPDT mediated by ALA, HAL, and HAL-ES at a concentration of 300 μM. The results showed that HAL-mediated aPDT killed approximately 66.7% of C. albicans cells, while neither HAL-ES- nor ALA-mediated aPDT produced significant antifungal effects. Since ALA is a water-soluble photosensitizer, it enters cells through active transport and drug uptake depends on energy, while lipophilic HAL enters cells through simple diffusion. Therefore, ALA is less efficient than HAL in penetrating cells to mediate aPDT effects (16, 17). For this reason, it failed to induce a potent aPDT effect under our experimental conditions, which was consistent with previous research (18, 19).

### HAL-ES promoted the healing of cutaneous C. albicans infection.

**FIG 8:**
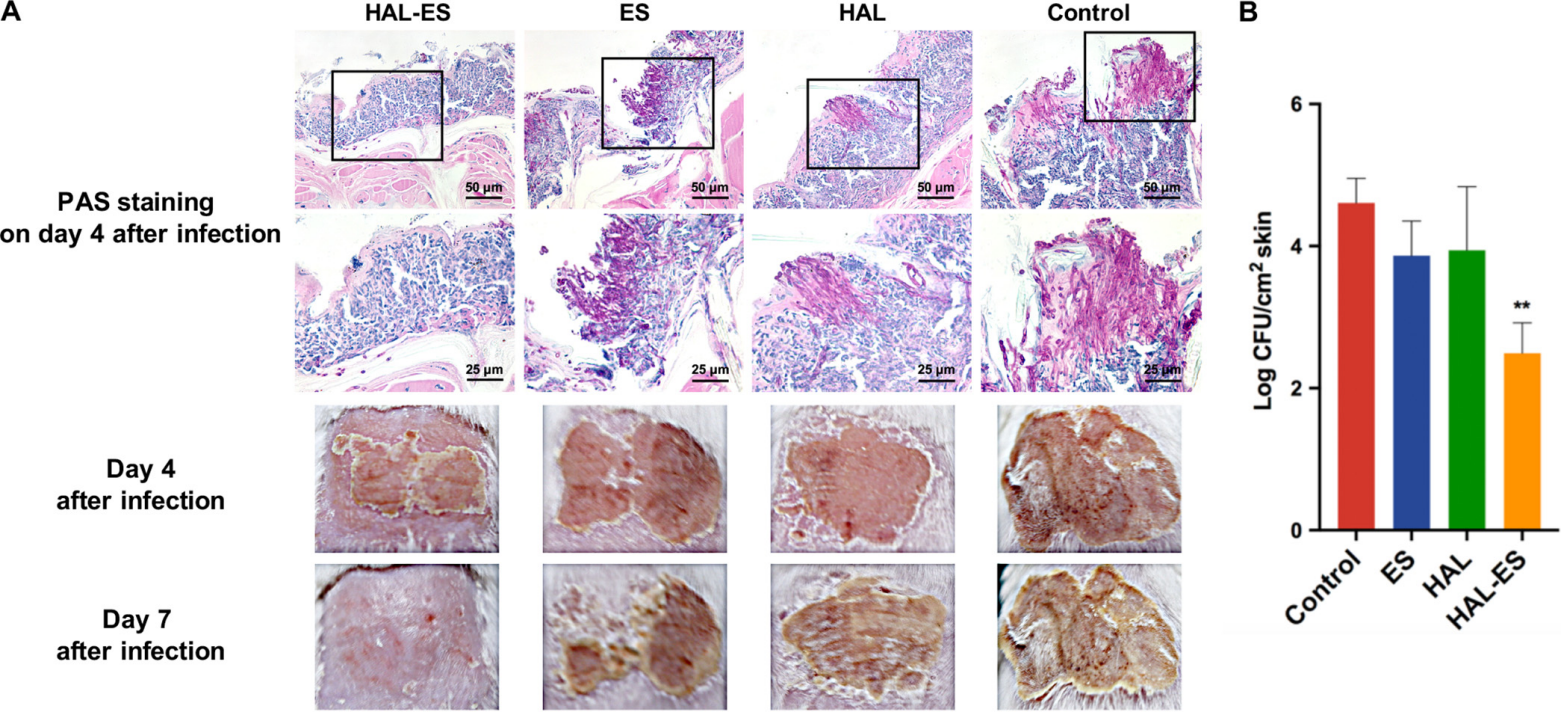
Effects of HAL-ES on cutaneous Candida albicans infection in mice. After 2 days of infection, the mice were treated with HAL-ES (containing 30% ES and 20 mM HAL), 20 mM HAL, ES, or PBS combined with aPDT. (A) Skin from infected mice harvested on day 4 after infection was sectioned and stained with PAS to highlight fungal cells. Hyphal forms can be observed penetrating the epidermis of mice in the HAL, ES, and control groups, while no hyphal forms were observed in the HAL-ES group. The appearance of skin infection was recorded on days 4 and 7 after infection. Images are representative of three independent experiments with at least six mice per group. (B) Fungal loads 4 days after infection (log CFU per square centimeter). Data are presented as means ± SD (*n* = 3). **, *P < *0.01 compared with the control group.

We evaluated the efficacy of topical administration in a mouse model of cutaneous candidiasis. Treatment was performed on day 2 postinfection. On the fourth day after infection, crusts were observed on the skin of mice in each group. Periodic acid-Schiff (PAS) staining showed that hyphae invaded the epidermis in the HAL, ES, and control groups, while no hyphal invasion was observed in the epidermis of mice in the HAL-ES group (Fig. 8A). On day 4 postinfection, the fungal load of the infected skin tissue homogenate was also significantly reduced in the HAL-ES group (Fig. 8B). On the seventh day after infection, the infected skin of mice in the HAL, ES, and control groups still had crusts attached, while the crusts on mice in the HAL-ES group had fallen off and the infection had resolved (Fig. 8A).

### HAL-ES inhibited protein translation through zinc restriction and downregulated expression of multidrug resistance-related genes.

**FIG 9:**
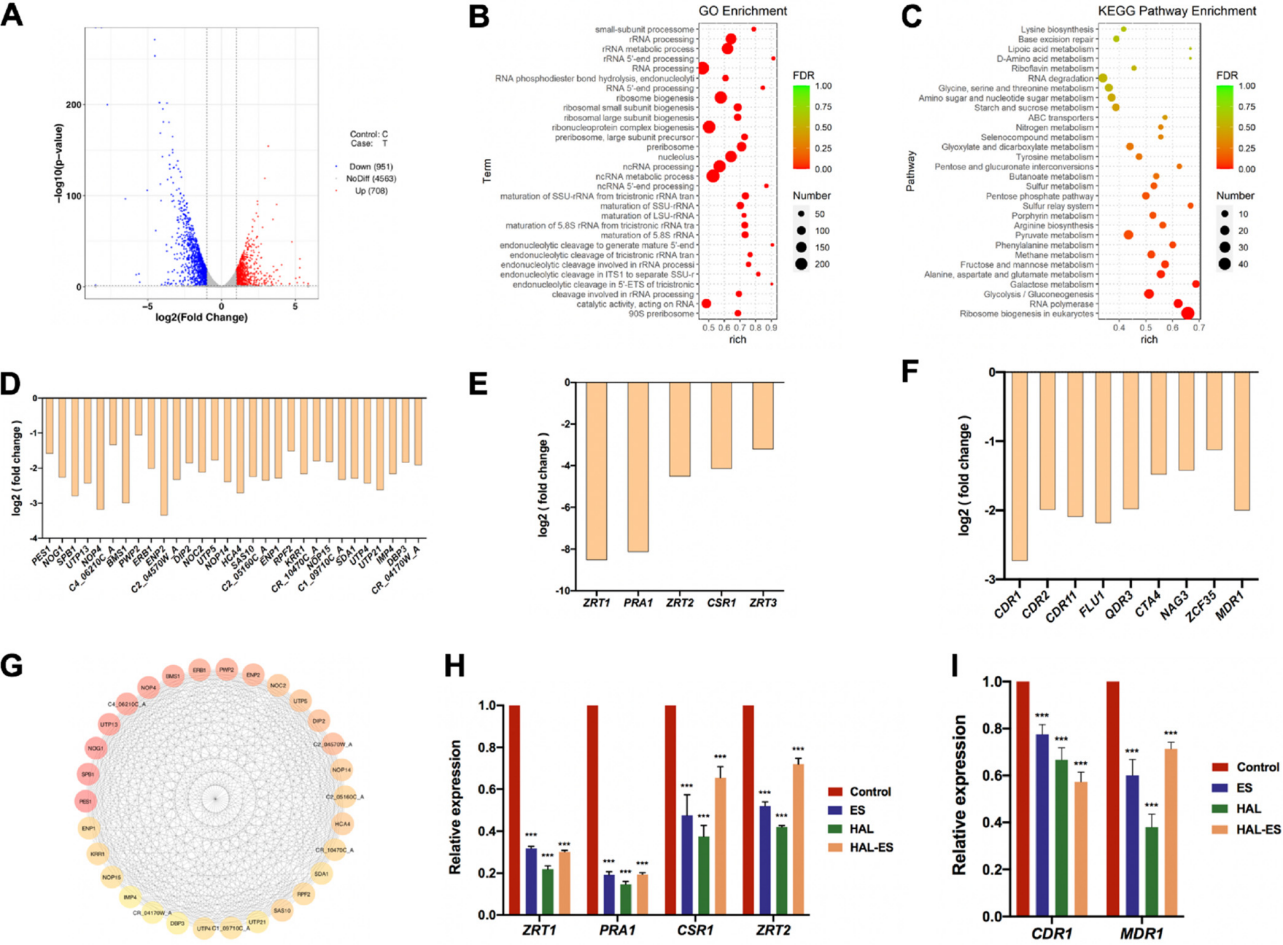
Transcriptome and qPCR analysis of Candida albicans biofilms over 24 h after HAL-ES treatment. (A) Upregulated (red) and downregulated (blue) genes. A |log_2_ fold change| of >1 was considered significant (*P < *0.05). (B and C) Gene Ontology (GO) and KEGG enrichment (C) analyses. The size of each point represents the number of differentially expressed genes (DEGs), and the color gradient from red to green represents the level of enrichment. (D to F) DEGs in the top 30 degrees of hub genes (D) or those associated with zinc homeostasis (E) or multidrug resistance (F). Significantly changed genes are shown, with decreasing *P* values (<0.0001) from left to right. (G) Top 30 degrees of hub genes based on the PPI network. (H and I) qPCR analysis of genes associated with zinc homeostasis (H) and multidrug resistance (I). C. albicans biofilms were treated with ES (30%), HAL (20 mM), or HAL-ES (containing 30% ES and 20 mM HAL) combined with aPDT. Data are presented as means ± SD (*n* = 3). ***, *P < *0.001 compared with the control group. FDR, false-discovery rate.

To further investigate the mechanism of HAL-ES antibiofilm activity, RNA-sequencing (RNA-seq) and quantitative PCR (qPCR) were used to determine the changes at the transcriptional level. The C. albicans biofilm of the treatment group was cultured for 24 h after HAL-ES-mediated PDT, as previously described (5). The C. albicans biofilm without drug treatment served as a control group. At 24 h after HAL-ES treatment of biofilms, 1,659 genes were differentially expressed. Of these, 708 were upregulated and 951 were downregulated (|log_2_ fold change| > 1; *P < *0.05) (Fig. 9A). The two genes with the most significant differences were zinc-regulated transporter 1 (*ZRT1*) and PH-regulated antigen (*PRA1*), which are both related to zinc homeostasis; their log_2_ fold changes were <–8. Other genes related to zinc homeostasis, such as zinc-regulated transporter 2 (*ZRT2*), *Candida* suppressor of *rok1* (*CSR1*), and zinc-regulated transporter 3 (*ZRT3*), were also strongly downregulated (Fig. 9E). In addition, the expression of multidrug resistance genes, such as *Candida* drug resistance 1 (*CDR1*), *Candida* drug resistance 2 (*CDR2*), *Candida* drug resistance 11 (*CDR11*), fluconazole resistance 1 (*FLU1*), quinidine drug resistance 3 (*QDR3*), *Candida* transactivating 4 (*CTA4*), *N*-acetylglucosamine 3 (*NAG3*), Zn(II)_2_Cys_6_ transcription factor 35 (*ZCF35*), and multidrug resistance 1 (*MDR1*), were significantly downregulated (Fig. 9F). The qPCR results were consistent with RNA-seq data and revealed that HAL played a more pronounced role in the downregulation of gene expression than ES (Fig. 9H and I).

Gene Ontology (GO) enrichment analysis was performed to characterize the cellular pathways affected by HAL-ES. We focused on the top 30 GO terms with the most differentially expressed genes (DEGs), all of which were associated with the C. albicans ribosome. In terms of molecular functions, catalytic activity acting on RNA was significantly enriched. In terms of biological processes, the categories of rRNA metabolic processes, ribosome biogenesis, and rRNA processing were notably enriched. At the cellular component level, nucleolus, preribosome, and large subunit precursors were significantly enriched (Fig. 9B). We utilized the Kyoto Encyclopedia of Genes and Genomes (KEGG) database to determine the signaling pathways related to the DEGs (Fig. 9C). The signaling pathways affected by HAL-ES included mainly those involved in ribosome biogenesis and RNA polymerase synthesis (false-discovery rate, <0.01). Other pathways also significantly affected by treatment included glycolysis and gluconeogenesis, galactose metabolism, fructose and mannose metabolism, and alanine, aspartate, and glutamate metabolism (false-discovery rate, <0.05), indicating that the treatment also changed the unique metabolic activity of biofilms (4). Based on the protein-protein interaction (PPI) network, the top 30 hub genes screened by degree had downregulated expression and were related to ribosome biogenesis (Fig. 9D).

In conclusion, these results suggest that HAL-ES reduces zinc homeostasis-related gene expression in biofilms, thereby inhibiting ribosome biogenesis and ultimately reducing C. albicans protein translation processes.

## DISCUSSION

Because our previous study described the effective antibiofilm activity of HAL-ES (5), this study further elucidated its mechanism and provided a theoretical basis for its clinical application in the treatment of candidiasis. HAL is a photosensitizer with antimicrobial effects (8), and ES is a nanovesicle carrier that also has antimicrobial activity in the presence of ethanol (20). We found that under biofilm-inducing conditions, HAL-ES inhibited the progression of various stages of biofilm formation, including early adhesion and hyphal formation and elongation in mid-development, as well as three-dimensional structure formation during maturation stages. Moreover, its antifungal effects were enhanced in comparison to those of fluconazole, HAL, or ES. Surprisingly, after only a brief exposure to HAL-ES, C. albicans showed a significant reduction in adhesion, hypha-forming ability, and fluconazole resistance. This is of great significance for the local application of HAL-ES in infected tissue, as it could prevent biofilm formation and the occurrence and development of invasive *Candida* infections. However, for yeast cultured in YPD medium, no significant differences were observed in the antifungal effects of ethanol, ES, or HAL-ES with the same ethanol concentration. Therefore, the antifungal effects varied under different culture conditions or C. albicans phenotypes. C. albicans was particularly sensitive to HAL-ES treatment under biofilm-inducing culture conditions.

At concentrations without antifungal effects, HAL-mediated aPDT was observed and was superior to that of ALA. HAL-ES did not mediate aPDT because the encapsulation of HAL by the phospholipids of ES and the penetration-promoting effect of ES were greatly attenuated by ethanol dilution. However, our previous study showed that, during the process of penetration, the ES vesicles were broken and the phospholipids were retained in the upper epidermis, with the drug gradually penetrating the lower skin layers (13). Therefore, we propose that HAL-ES is a candidate for the treatment of mucocutaneous candidiasis through HAL-mediated aPDT.

We used transcriptome sequencing to study the antibiofilm mechanism of HAL-ES and found that the expression levels of zinc homeostasis-related genes, such as *ZRT1*, *ZRT2*, *PAR1*, *CSR1*, and *ZRT3*, remained downregulated 24 h after HAL-ES treatment. Zrt1 and Zrt2 are major membrane transporters for zinc uptake, and their transcriptional levels are regulated by the zinc homeostasis regulator transcription factor Csr1. Pra1 is an extracellular zinc-binding protein that sequesters zinc from host cells and subsequently reassociates with the fungal cell surface zinc transporter Zrt1 (21). Zrt3 mediates the efflux of zinc from the vacuole, which is responsible for zinc storage (22). Therefore, the uptake, storage, and utilization of zinc in C. albicans biofilms can be severely impaired by HAL-ES. A previous study found that simple zinc chelators only inhibited the growth of C. albicans, with concentrations well above the MIC required to produce a fungicidal effect (23). However, we found that the MIC and MFC were similar for HAL-ES because, compared to a zinc chelator, it caused an overall reduction in the uptake, storage, and utilization of zinc by C. albicans. This is because HAL-ES contains two components, HAL and ES, that result in multiple antibiofilm targets.

Impairment of zinc utilization results in the inhibition of C. albicans protein translation (23, 24), which explains the significant inhibition of biofilm growth and pathogenicity following HAL-ES-mediated treatment in our previous research (5). Approximately 8% of yeast proteins require zinc for their activity (25), especially enzymes related to pathogen survival and virulence as well as zinc finger transcription factors involved in transcriptional regulation (22, 24). Previous studies on bacteria demonstrated that zinc deprivation inhibits ribosome biogenesis and translational activity, thereby reducing growth and pathogenicity (26). Live vaccine strains with significant attenuation and immunogenicity have been developed by knocking out the gene encoding the bacterial zinc transporter ZnuABC (26). In C. albicans, Pra1, Zrt1, and Csr1 contribute to hyphal growth, biofilm formation, and virulence (21, 27–29). Csr1 regulates the balance of yeast and hyphal cells in biofilms (28). Pra1 mediates endothelial cell damage by scavenging host zinc and promotes immune escape by regulating host complement attack at the C3 level (30, 31). *ZRT2* knockout resulted in reduced virulence in a mouse model of candidiasis (32). Therefore, by downregulating the expression of genes related to zinc homeostasis in C. albicans, HAL-ES can reduce its morphological transition, biofilm formation, pathogenicity, and immune escape.

Zinc deprivation in C. albicans by HAL-ES tends to be selective, because the core fungal zinc uptake and regulation mechanisms are evolutionarily conserved and there is little homology between fungal and human zinc transporter gene sequences (24, 33). Our further experiments in a mouse model of cutaneous candidiasis confirmed the superior efficacy of HAL-ES *in vivo*. Our results provide evidence for the clinical application of HAL-ES.

Interestingly, we also demonstrated that HAL-ES increased the susceptibility of C. albicans to fluconazole after a brief incubation. This suggested that the increased fluconazole sensitivity of biofilms in our previous study (5) was not only due to the direct antibiofilm effect of HAL-ES. However, ethanol, ES, or HAL alone was ineffective, indicating a synergistic sensitization effect among the components of HAL-ES. RNA-seq and qPCR showed that the expression of the multidrug-resistant efflux pump genes *CDR1* and *MDR1* (34) was downregulated after biofilm treatment with HAL-ES. This indicated that HAL-ES could increase drug susceptibility by reducing drug efflux.

Traditional antifungal drugs, such as azoles, polyenes, and echinocandins, mainly target cell wall and membrane metabolism. In bacteria, ~50% of antibiotics act on translation processes (35). Although protein synthesis pathways are not often targeted in antifungal research, our results suggest that HAL-ES could inhibit fungal protein synthesis by targeting zinc homeostasis, thus producing superior antibiofilm activity. A limitation of our study is that only one standard C. albicans strain, SC5314, was tested, but the results of this study are expected to be representative, since SC5314 has been widely used as a reference strain in C. albicans biofilm research (36–39).

Collectively, the results of this study have revealed the antibiofilm mechanism of HAL-ES and demonstrated its potential application in the treatment of related fungal infections, especially drug-resistant and refractory infections caused by biofilms. We have also uncovered a new target for the treatment of biofilm-related infections by zinc restriction.

## MATERIALS AND METHODS

### Strains, growth conditions, and reagents.

C. albicans strain SC5314 was maintained on YPD plates (0.5% yeast extract, 1% Bacto-peptone, 2% glucose, and 1.4% agar) at 37°C. HAL-ES (containing 30% ES and 20 mM (5 mg/ml) HAL; NMT Biotech, Suzhou, China) and blank ES without HAL (containing 30% ethanol) were prepared as previously described (5). HAL and ALA solutions were prepared in double-distilled water and filter sterilized.

### Determination of the MIC and MFC.

As recommended by the Clinical and Laboratory Standards Institute, an antifungal susceptibility test was conducted using the broth dilution testing reference method M27-A3 (40, 41). HAL-ES and ES were tested using a 0.03 to 15% ethanol concentration range. Fluconazole (0.125 to 64 μg/mL; Macklin, Shanghai, China) was included as a positive control. The MIC was defined as the lowest concentration that showed no visible fungal growth and turbidity after 24 to 48 h of incubation. We assessed the growth of C. albicans 36 h after treatment under an inverted microscope (IX73; Olympus, Tokyo, Japan). Image acquisition was conducted using cellSens Dimension software (Olympus). Subculturing of 100 μL of the culture from each optically clear well was performed on YPD plates at 37°C for 24 to 48 h to obtain the MFC, the lowest drug concentration at which there was no fungal growth. Experiments were performed three times independently.

### Time-kill assay.

The time-kill assay was performed according to previously described methods, with minor alterations (41). C. albicans cells (1 × 10^6^ CFU/mL) were incubated in 5 mL of YPD medium containing ethanol, ES, HAL, or HAL-ES at 37°C with constant shaking (200 rpm). C. albicans cells in drug-free YPD medium were used as controls. At the desired time points (0, 1, 3, 6, 12, and 24 h), a 100-μL aliquot was removed for each test condition and serially diluted (10-fold) in saline (0.9% NaCl), after which 5 μL of each dilution was spread on a YPD plate. The colony count on each YPD plate was determined after incubation at 37°C for 24 to 48 h. Experiments were performed three times independently.

### Cell adhesion assay.

The cell adherence assay was performed according to previously reported methods, with minor alterations (42–44). RPMI 1640 (Thermo Fisher Scientific, Waltham, MA, USA) or YPD medium were prepared containing HAL-ES, HAL, ES, ethanol, or fluconazole. An overnight culture of C. albicans was adjusted to a final concentration of 1 × 10^6^ CFU/mL in drug-containing RPMI 1640 or YPD medium. For drug-exposed cells, C. albicans was incubated in drug-containing YPD medium at 37°C with constant shaking (200 rpm) for 2 h, then washed thrice with phosphate-buffered saline (PBS) to ensure complete drug removal, and finally resuspended in drug-free RPMI 1640 medium. For drug-containing cells, C. albicans was directly resuspended in drug-containing RPMI 1640 medium. Next, we added 1 mL of the drug-containing or drug-exposed cell suspension in RPMI 1640 medium to each well of a 24-well plate. The C. albicans cell suspension was incubated in drug-free medium as a control. After 2 h of incubation, the medium was removed, and nonadherent cells were removed by washing the wells thrice with PBS. After methanol fixation, C. albicans cells were stained with 0.1% crystal violet and dried overnight. Cells were observed under an inverted microscope (IX73; Olympus). Image acquisition was conducted using cellSens Dimension software (Olympus). Experiments were performed three times independently.

### Yeast-to-hypha morphogenesis.

The effects of drugs on the yeast-to-hypha morphogenesis of C. albicans were measured according to previously described methods, with minor alterations (43, 45). RPMI 1640 medium containing HAL-ES, HAL, ES, ethanol, or fluconazole were prepared. For drug-exposed cells, C. albicans was incubated in drug-containing RPMI 1640 medium at 37°C with constant shaking (200 rpm) for 1 h, then washed thrice with PBS to ensure complete drug removal, and finally resuspended in drug-free RPMI 1640 medium supplemented with 10% fetal bovine serum (FBS). For drug-containing cells, C. albicans was directly resuspended in drug-containing RPMI 1640 medium supplemented with 10% FBS. C. albicans hyphae formation was induced by continuous shaking at 200 rpm for 4 h at 37°C. Subsequently, the culture was washed thrice with PBS and observed under a microscope (IX73; Olympus). Image acquisition was conducted using cellSens Dimension software (Olympus). Grayscale conversion, contrast, and brightness of the image were adjusted using Adobe Photoshop 20.0.7 (Adobe Systems, CA, USA). The C. albicans cell suspension was incubated in the drug-free medium as a control. Experiments were performed three times independently.

### Biofilm formation.

As previously reported (42, 44, 45), C. albicans cells were allowed to adhere for 2 h, after which RPMI 1640 medium containing HAL-ES (7.5% ES and 5 mM HAL), ES (7.5%), ethanol (7.5%), or fluconazole (1 μg/mL) was added. After incubation at 37°C for 24 h, the biofilms were stained with crystal violet and observed under an inverted microscope (IX73; Olympus). Image acquisition was conducted using cellSens Dimension software (Olympus). Experiments were performed three times independently.

### Antifungal sensitivity assay.

The sensitivity of the C. albicans cells to fluconazole was evaluated using a disk diffusion assay as described in National Committee for Clinical Laboratory Standards document M44-P (46). Briefly, C. albicans cells were grown in the absence or presence of drugs for 2 h in YPD medium at 37°C. The cultures were inoculated on Mueller-Hinton agar plates (Hopebio, Qingdao, China) supplemented with 2% glucose and methylene blue (0.5 μg/mL). Fluconazole (25 μg) disks (BKMAM, Hunan, China) were placed in the center of the plates, which were then incubated for 24 h at 37°C, and the zone of clearance was recorded (34, 45). An image of each petri dish was recorded using a digital camera (XT2; Fujifilm, Tokyo, Japan). Experiments were performed three times independently.

### aPDT of C. albicans.

Antifungal-free concentrations of ALA, HAL, and HAL-ES against C. albicans were first determined by incubation in YPD or RPMI 1640 medium for 24 h in the dark as described above (40, 41). In YPD medium, C. albicans colonies were counted after 4, 12, and 24 h of coincubation with ALA, HAL, or HAL-ES. In RPMI 1640 medium, C. albicans cells were coincubated with ALA, HAL, or HAL-ES for 24 h, and the growth of C. albicans was observed under an inverted microscope (IX73; Olympus, Tokyo, Japan). Image acquisition was conducted using cellSens Dimension software (Olympus). C. albicans cells in drug-free medium were used as controls. After determining the drug-free antifungal concentration of 300 μM, the effect of aPDT was analyzed as previously described (5). C. albicans cells were incubated with 300 μM HAL-ES, HAL, ALA, or PBS for 30 min at 37°C in the dark and then rinsed twice with PBS to remove the drug solution and resuspend the cells. The cells were immediately irradiated using a light-emitting diode (LED) source (LED-IB PDT; Wuhan Yage Optic and Electronic Technique, Wuhan, China) for 30 min (wavelength range, 633 ± 10 nm; power, 60 mW/cm^2^; irradiation distance,10 cm). A total volume of 10 μL of serially diluted cell suspension was spread on YPD agar plates, and colonies were counted after 24 to 48 h of incubation at 37°C. Experiments were performed three times independently.

### Treatment of skin candidiasis.

We used 4‐ to 6-week-old female BALB/c mice (Laboratory Animal Center of Nanfang Hospital, Guangzhou, China) in this study. The study design was approved by the Institutional Animal Care and Use Committee of Nanfang Hospital, Southern Medical University (NFYY-2021-1033). All protocols adhered to the Principles of Laboratory Animal Care guidelines of the Laboratory Animal Center of Southern Medical University. Skin candidiasis was induced as previously described (47). Briefly, mice were anesthetized with a mixture of ketamine and xylazine (100 mg and 10 mg/kg body weight, respectively), and their back hair was removed with electric clippers and a depilatory cream (Veet, Shanghai, China). The stratum corneum was removed with 15 strokes using 150-grit sandpaper. After washing with sterile PBS, 1 × 10^7^ C. albicans cells in 50 μL of PBS were applied to the skin. After 2 days, the infected skin was incubated with HAL-ES (containing 30% ES and 20 mM HAL), 20 mM HAL, ES, or PBS for 30 min in the dark and then exposed to a 60-mW/cm^2^ LED light for 30 min. The skin condition was observed and recorded daily using a digital camera (XT2; Fujifilm, Tokyo, Japan). On day 4 after infection, mice were euthanized by cervical dislocation, and a 2.0-cm^2^ section of infected skin was excised and divided into two pieces. One part was subjected to PAS staining and then examined under a microscope (BX51; Olympus). Images were processed using DP Controller and DP Manager software (Olympus). The other part of the skin sample was homogenized in sterile PBS containing penicillin and streptomycin and then spread on YPD plates. Colony counts were obtained 24 to 48 h later. Data are representative of three independent experiments with at least six mice per group.

### RNA-seq analysis of C. albicans biofilms.

After the C. albicans biofilms were treated with HAL-ES-mediated aPD as previously described (5), they were further incubated in RPMI 1640 medium at 37°C for 24 h, after which the cells were harvested for transcriptomic analysis. C. albicans biofilms without drug treatment served as a control group. Total RNA extraction, RNA-seq, and bioinformatic data collection were performed by Shanghai Personalbio (Shanghai, China) (48). Each group was analyzed in triplicate samples.

### Gene expression analysis by qPCR.

After the C. albicans biofilms were treated with HAL-ES, HAL, or ES-mediated aPD as previously described (5), they were further incubated in RPMI 1640 medium at 37°C for 24 h. The control group was treated with PBS. C. albicans biofilm cells were collected, and total RNA was isolated using an EASY-spin total RNA extraction kit (catalog number RN10; Aidlab Biotechnologies, Beijing, China). cDNA was obtained using a reverse transcription kit (catalog number RR036A; TaKaRa Bio, Beijing, China), and qPCR was performed using SYBR Premix *Ex Taq* II (catalog number RR820A; TaKaRa Bio) on a QuantStudio 5 real-time PCR system (Thermo Fisher Scientific). Primers used for qPCR were synthesized by Tsingke Biological Technology (Beijing, China) (49, 50), and their sequences are listed in Table S1. qPCR data were normalized using the geometric mean of *RIP* and *LSC2* as internal reference genes (51). The average relative gene expression was quantified using the 2^−△△^*^CT^* method and data from three independent experiments.

### Statistical analysis.

One-way analysis of variance or Student's *t* test (two-tailed, unequal variance) was used to assess the statistical significance of the differences between the treatment and control groups. All results were obtained from three independent experiments, and each value was expressed as the mean ± standard deviation (SD). A *P* value of <0.05 was considered significant.

### Data availability.

The RNA-seq data from this study have been deposited in the NCBI database under BioProject accession number PRJNA856036.
