# Supplementary material for: Hexyl-Aminolevulinate Ethosomes: a Novel Antibiofilm Agent Targeting Zinc Homeostasis in Candida albicans
Source: Microbiol Spectr. 2022 Oct 27;10(6):e02438-22. doi: 10.1128/spectrum.02438-22 (PMC9769717; doi:10.1128/spectrum.02438-22)
Supplement: Supplemental file 1 — Table S1. Download spectrum.02438-22-s0001.pdf, PDF file, 0.2 MB [file spectrum.02438-22-s0001.pdf]

SUPPLEMENTAL MATERIAL

Title: Hexyl-Aminolevulinate Ethosomes: A Novel Antibiofilm Agent Targeting Zinc Homeostasis in *Candida albicans*

Authors: Yingzhe Wang, Wei Long, Feiyin Zhang, Meimei Zhang, Kang Zeng, Xiaoliang Zhua

TABLE S1. Sequences of primers used in this study

Gene symbol	Primers (5′ to 3′)	Source
ZRT1	F: GCTTCTACAATTACCACACCACC<br>R: GGAACTGGCAAGATTGAACCT	(1)
ZRT2	F: ACTGGGCCAATAACAGAATACC<br>R: GGATATGGATTGGCATCAGCT	(1)
PRA1	F: TAGCTGAACATGCCAGGGACC<br>R: CAATAACCAGCCCAGCCATC	(1)
CSR1	F: ACTTTCCCATTATGACGTTCACAC<br>R: GCAAACCCAAAGTGAAGAAAGAGG	(1)
CDR1	F: ACTCCTGCTACCGTGTTGTTATTG<br>R: ACCTGGACCACTTGGAACATATTG	(2)
MDR1	F: AGTTGCTTGGGGTAGTTCCG<br>R: CTTGCTCTCAACTTTGGTCCG	(3)
RIP	F: TGTCACGGTTCCCATTATGATATTT<br>R: TGGAATTTCCAAGTTCAATGGA	(4)
LSC2	F: CGTCAACATCTTTGGTGGTATTGT<br>R: TTGGTGGCAGCAATTAAACCT	(4)

REFERENCES

1. Duan X, Xie Z, Ma L, Jin X, Zhang M, Xu Y, Liu Y, Lou H, Chang W. 2022. Selective Metal Chelation by a Thiosemicarbazone Derivative Interferes with Mitochondrial Respiration and Ribosome Biogenesis in Candida albicans. Microbiol Spectr 10:e01951–21.

2. Jia W, Zhang H, Li C, Li G, Liu X, Wei J. 2016. The calcineruin inhibitor cyclosporine a synergistically enhances the susceptibility of Candida albicans biofilms to fluconazole by multiple mechanisms. BMC Microbiol 16:113.

3. Maheronnaghsh M, Teimoori A, Dehghan P, Fatahinia M. 2022. The evaluation of the overexpression of the ERG-11, MDR-1, CDR-1, and CDR-2 genes in fluconazole-resistant Candida albicans isolated from Ahvazian cancer patients with oral candidiasis. J Clin Lab Anal 36:e24208.

4. Nailis H, Coenye T, Van Nieuwerburgh F, Deforce D, Nelis HJ. 2006. Development and evaluation of different normalization strategies for gene expression studies in Candida albicans biofilms by real-time PCR. BMC Mol Biol 7:25.
